# Marching for the myth of science: A self‐destructive celebration of scientific exceptionalism

**DOI:** 10.15252/embr.201744935

**Published:** 2017-08-18

**Authors:** Bart Penders

**Affiliations:** ^1^ Department of Health, Ethics and Society Care and Public Health Research Institute (Caphri) Maastricht University Maastricht The Netherlands

**Keywords:** S&S: History & Philosophy of Science, S&S: Politics, Policy & Law

## Abstract

The Marches for Science this April celebrated the unique role of science as well as nerd culture. But their portrayal of science's exceptionalism and elitism did little to convince citizens wary of how science affects politics and their lives.

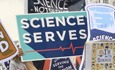

US President Donald Trump has quickly become a major source of concerns and annoyance among scientists since he took office last year. His various actions and announcements—from barring scientists from various Muslim countries from entering the USA to his proposed budget cuts for research to his personnel decisions for federal agencies—have prompted scientists to take it to the streets in unprecedented numbers. From Washington DC to Busan, South Korea and from Sydney, Australia to Berlin, Germany, between 300,000 and 500,000 participants at the March of Science shouted slogans and placated posters on Earth Day, April 22 (Fig 1). While President Trump's rhetoric and policies provided the stimulus, a general feeling that science has become irrelevant in politics, whether in the heat of political campaigns, or in the day‐to‐day governing of a country, further fuelled and supported scientists’ engagement.

**Figure 1:**
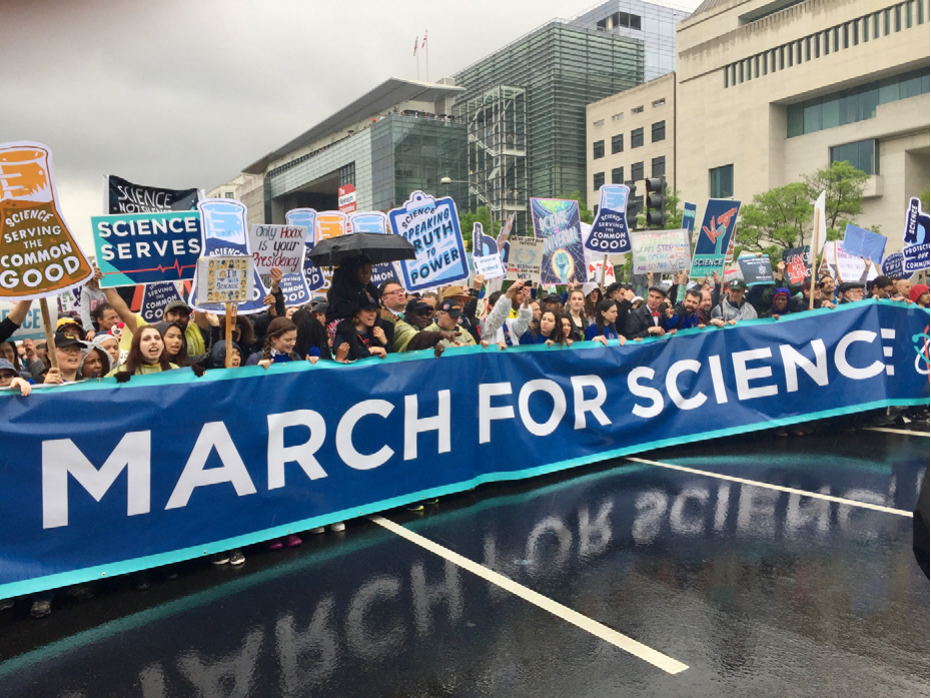
March for Science in Washington, DC, USA, 22 April 2017 Image by Paul Becker, Grove City, OH, USA, under the terms of a CC‐BY license. The content of the article does not necessarily represent the photographer's opinion.

The Trump administration has not just regarded science as merely inconvenient but taken an openly hostile attitude to research that does not fit into its political agenda.

Yet, Trump is not the first US president to draw the ire of scientists. US President George W. Bush was perceived as the anti‐science president given his scepticism of climate change, his tolerance for teaching intelligent design in school and his general disdain for scientific advice: science was “an annoying inconvenience to its political agenda; an inconvenience that needed to be ignored, suppressed or even manipulated for political purposes” 1. Throughout both of President Bush's terms, scientists argued and voiced their concerns. However, none of those protests and arguments ever rose to the scale of the March for Science on April 22.

## “Make America Smart Again”

The Trump administration has not just regarded science as merely inconvenient but taken an openly hostile attitude to research that does not fit into its political agenda. The radical policies and extreme cutbacks to research his administration proposed after inauguration, as well as the aggressive rhetoric, unreliable demeanour and continued talk of so‐called alternative facts have created a radically different atmosphere. This stimulated scientists and sympathisers in the USA to take it to the streets in larger numbers than ever before. Scientists from other countries joined the initiative, often addressing their own politicians’ dismissal of science. During last year's Brexit campaign in the UK, for instance, then Secretary of State for Justice Michael Grove dismissed the advice from expert bodies—including the IMF—with “the people have had enough of experts”.

## “I can't believe I'm protesting for reality”

One of the initiators of the March for Science acknowledged that current political events were a key trigger, yet argued that her motivation mostly comes from “the subtle and steady debasing of science over decades” 2. This debasing, or, in different terminology, the perception of loss of political or public credibility, is a common concern among scientists and was an important rationale for the March for Science. Its mission, listed on its website, is “a celebration of science”, speaking out against “discrediting scientific consensus and restricting scientific discovery” and “encouraging the application of science in policy”. A survey of more than 1,000 people, who intended to march, showed that their main motivation to participate were “encouraging policies based on science; encouraging the public to support science; opposing political attacks on science; and protesting cuts in science funding” (www.sciencebusiness.net/news/80236/View-from-the-US-marchers-want-to-promote-and-defend-science).

… debasing, or, in different terminology, the perception of loss of political or public credibility, is a common concern among scientists and was an important rationale for the March for Science.

The March of Science therefore seems like a natural reaction to the “war on science”: the increasingly hostile attitude of politicians and politics against scientists and their expertise. The willingness of so many scientists to stand up for science and to become politically active—even though many vigorously deny this—is certainly laudable. However, I argue that scientists actively coming to the defence of science‐based policy, defending free inquiry, funding and autonomy, and attempting to promote an image of science as neutral are becoming political actors themselves. They especially can no longer uphold an image of science as neutral and free from values or deny the close ties between science, politics and business. It also means that others might see their protests as an attempt by an elite to protect their privileges. In fact, many scientists decided not to attend, even if they support the cause, because of the risk of politicisation of science, and thereby undermining the March's goals.

## “Science is not a liberal conspiracy”

In an excellent critique of the war on science, Miller argues that the war is real, but it is rather a war on big government, and science, through its increasingly close relationship with political or economic power, has become collateral damage 3. Thus, a strategy to combat big government, which is a long‐term goal of US Republicans and conservatives, will inevitably target the instruments that grant government power over its constituents, including science: “They attack science's forms of truth‐making, its databases, and its budgets not out of a rejection of either science or truth, but as part of a coherent strategy to weaken the power of the federal agencies that rely on them” 3. Protesting against budget cuts or against governments encroaching upon the territory scientists claim is theirs, can therefore not be detached from political realities. Protesting against budget cuts inevitably means protesting against the government and thereby opens rhetorical avenues to paint science as a partisan issue. As Mervis wrote: “For Congress, the March for science is a democratic event” (www.sciencemag.org/news/2017/04/congress-march-science-democratic-event).

Science is inseparable from politics to the point that science itself becomes a form of power. We should therefore see the war on science—if it exists at all—or the public distrust of experts in a context of public discontent with authorities that exert power over citizens’ lives. In his excellent critique of populism, Van Reybrouck 4 argued that populist politics are merely a symptom of an underlying problem, namely that a significant portion of citizens no longer feel represented by elected officials, and in fact, no longer are represented. Following Miller's argument above, such politics cannot be separated from the scientific infrastructure, as it helps to facilitate this power. Many populist political campaign across the USA and Europe—most notably Trump's campaign—have voiced displeasure with the *status quo*, and, as a consequence, of the scientific experts and expertise that are part of it. Rejecting science is therefore a way to rebel against the political establishment.

## “Expert is not a dirty word”

Preventing that science and its institutions become collateral damage in this rebellion would require recognising that science is not an isolated cultural activity but actively shapes politics and society, as well as is actively shaped by them. Such a growing understanding that science is neither pure nor neutral, that the boundaries between society and science are impossible to pin down, that norms and values inform research and that scientists are people too, is the legacy of decades of research in the history, philosophy, anthropology and sociology of science. Sadly, the dominant tone of the Marches for Science pointed elsewhere, towards resurrecting a myth of science as a disconnected, objective enterprise.

Every scientist participating in the March, protesting budget cuts and eroding authority, knows well that research is work, that it is about securing resources, about managing people and about publishing to achieve tenure and grants—in short, when it comes to managing daily work, science is not that different from business or government. In reference to science, business and government, Miller rightly asserts that “[n]one of the three can any longer pretend that they stand outside politics” 3. To accept or to embrace the politics and responsibility of science therefore requires actively denying an image of scientific exceptionalism. Science is an important and highly valued part of our culture. It generates both solutions and problems, it is most certainly not always right, and it is just as (im)perfect as all other human activity. Scientists know this, yet they choose to hide or downplay this when engaging publics and politics.

## “Listen to your nerds”

Embracing its political character instead would enable scientists to stress the ability of science to shape the world, and thereby find and secure public support. However, many commentators, organisers and participants urged the protestors to focus on the image of science, not its politics: “[a] political stance affords little long‐term gain for scientists or the public. Let's march for public outreach and understanding of science […]” 5. While most marches saw anti‐Trump signs and rhetoric and some of the organisers have chosen to embrace the political context in which science lives, most protesters chose to defend science advocacy or science in general as shown by their slogans, signs and chants. While it may appeal to a purist or ideal image of science, it paradoxically represented the political realities. Many signs reaffirmed the scientific method, the ability to discover truths and the privileged ability of scientists to access and produce knowledge: “You think your anecdote trumps my meta‐analysis? How quaint”. Similarly, the March for Science website and a plethora of posts insist on keeping politics out of science, but not the other way around: “science‐based policy, not policy‐based science”. Of course, protesters’ signs are intended to send clear messages with little room for nuance. However, the characterisation of science at the marches stressed its apolitical character, its ability to produce value‐free truth and its superiority over other ways of understanding reality.

…a strategy to combat big government […] will inevitably target the instruments that grant government power over its constituents, including science…

References to truth, neutrality, objectivity and the value‐free character of science are also abundant on March for Science websites, Facebook and in opinion pieces in the scientific literature. For example, Rudder writes “Scientists’ findings deserve respect specifically because they emanate from procedures that ensure neutrality” 5. The American Association for the Advancement of Science (AAAS) openly supported the march “to protect the rights of scientists to pursue and communicate their inquiries unimpeded, expand the placement of scientists throughout the government, build public policies upon scientific evidence, and support broad educational efforts to expand public understanding of the scientific process” (www.aaas.org/news/aaas-and-march-science-partner-uphold-science). A focus on method and procedures, however, obscures all the ways in which science has become increasingly entangled with government and business. Faust, in *Slate* Magazine, therefore described the March as a “cringe‐worthy hive‐mind mentality” celebrating a science in such vague terms that “our culture's understanding of science is very, very broken, and on Saturday, it was impossible to ignore” 6.

Embracing its political character instead would enable scientists to stress the ability of science to shape the world, and thereby find and secure public support

He is not the only one expressing his worries about the March for Science in the context of the relationship between scientists and public, or between protesters and audiences. Whereas Faust discusses the public's misunderstanding of what science is, or ought to be, others focus on how the March for Science may influence public views or public credibility of experts. Mervis, for instance, cites Lubell to remember that many people view scientists as members of the very establishment that a significant number of citizens are currently resisting or opposing (www.sciencemag.org/news/2017/01/science-march-planners-here-s-some-unsolicited-advice). In a context in which scientists are regarded as privileged individuals and science as an elite activity, the March for Science, being a political rally after all, has a tough job reaching out to the general populace.

Two of the four primary motivations for participants to join the March for Science were encouraging science‐based policies, and public support for science. Explicit celebrations of geek culture and signs boasting scientific puns are of course hilarious and, to most of us, recognisable. As inside jokes, their ability to reach out to non‐scientists is however limited. While funny signs suggest science's exceptionality, AAAS’ statement is quite clear: scientists want to be left alone, they want influence without being influenced and “want support for instructing—not involving—the public in the scientific process, a greater influence on policymaking, and no political accountability” 7. As such, the marches were a celebration of exceptionalism, elitism and, intentional or not, an attempt to propagate the myth of science as the supplier of truths free of bias. On the eve of the March for Science, Carrol expressed the hope that scientists would “March for the right to be wrong” 8. It turned out to be less reflective.

## “Are marches effective? Ask a sociologist”

The March for Science was neither the exclusive domain of engineers and scientists, nor are all commenters, participants or organisers unaware of these issues. Holznienkemper 9 already stressed the relevance of values and normative frameworks in, for instance translating scientific results into policy. The “secret” Facebook group “March for Science” counted more than 800,000 members. Some are actively participating in politics and are increasingly accepting that their vocation is indeed social and political. Among the hundreds of posts on the Facebook group calling for more funding and science education are also reflective comments, wondering about the tone of the protest, ways to merge different ways of knowledge making and more. For instance, Jamie Tarich observed that “[W]hen it comes to critiquing anyone who disagrees [with us], all scientific standards and expectations get thrown out the window”.

… the characterisation of science at the marches stressed its apolitical character, its ability to produce value‐free truth, its superiority over other ways of understanding reality

Following April 22^nd^, new budget proposals, continued anti‐science rhetoric from the White House and the Trump administration's decision to leave the Paris Climate Accord, shows that the debasing of science that sparked the march remains unchanged. Rather than being effective, this brief analysis of the arguments, support, positions, chants and signs at the marches suggests that the March for Science was a step backwards for science in terms of political self‐awareness, reflective capacity and, as a consequence, its ability to exert power.

The Marches for Science were spectacular, great fun, well organised and, probably, futile. The scientists were media‐savvy, celebrated their geek culture and made abundant use of humour, which is always good medicine in controversial debates. Humour can be used to entertain and to build communities but it can also reinforce exceptionalism through inside‐only puns. What the Marches did not do was using humour to build trust using self‐mockery or showing humility. Ultimately, scientists were not marching to reach out to the general public, particularly those who are displeased with how policies and technology encroach on their lives, but to defend and portray a mythical image of science. The image they did portray was of science as an elitist, entitled, detached and naïve enterprise.

Ultimately, scientists were not marching to reach out to the general public, […] but to defend and portray a mythical image of science.

A common sign at every march proclaimed that “Science cannot be silenced”. Yet, a science that aims to exert influence through the production of value‐free truths without outside interference is easily silenced. All one has to do is stop listening. Thankfully, society has not stopped listening because that form of science is, as Daniel Sarewitz calls it, “a beautiful lie” 10. The fact that science cannot be separated from our culture is its key strength. It is what makes science relevant, almost omnipresent and always tangible. It makes science social, political and moral—just like everything else. It is also the true reason why science cannot be silenced.

## Conflict of interest

The author declares that he has no conflict of interest. He did not participate in his local March for Science for the reasons explained here.
